# The E-modulus of the oocyte is a non-destructive measure of zona pellucida hardening

**DOI:** 10.1530/REP-21-0122

**Published:** 2021-07-28

**Authors:** Carlo Schmitz, Seyedeh Zeynab Sadr, Hagen Körschgen, Michael Kuske, Jennifer Schoen, Walter Stöcker, Willi Jahnen-Dechent, Julia Floehr

**Affiliations:** 1 Helmholtz-Institute for Biomedical Engineering, Biointerface Laboratory, RWTH Aachen University Medical Faculty, Aachen, Germany; 2 Institute of Molecular Physiology, Cell and Matrix Biology, Johannes Gutenberg-University Mainz, Mainz, Germany; 3 Leibniz Institute for Farm Animal Biology, Institute of Reproductive Biology, Dummerstorf, Germany

## Abstract

After fertilization, the oocyte-specific metalloproteinase ovastacin is released and cleaves the zona pellucida protein 2 (ZP2), making the zona pellucida impermeable to sperm. Before fertilization, the zona remains permeable because previously released ovastacin is inhibited by fetuin-B. Consequently, in the absence of fetuin-B, ZP2 cleavage occurs prematurely and leads to infertility of female fetuin-B deficient mice. In contrast, fetuin-B/ovastacin double-deficient oocytes show a permanently permeable zona with intact ZP2. In this study, we asked if the elastic modulus of the zona pellucida informs about ZP2 cleavage and thus could serve as a new reference of oocyte fertility. Therefore, we determined the elastic modulus of mouse oocytes by nanoindentation as a direct measure of mechanical zona hardening. The elastic modulus reflects ZP2 cleavage, but with more than double sensitivity compared to immunoblot analysis. The elastic modulus measurement allowed to define the range of zona hardening, confined by the extreme states of the zona pellucida in fetuin-B and ovastacin-deficient oocytes with cleaved and uncleaved ZP2, respectively. We present here nanoindentation as a method to quantify the effect of potential contributing factors on the zona hardening of individual oocytes. To demonstrate this, we showed that mechanical hardening of the zona pellucida is forced by recombinant ovastacin, inhibited by additional administration of fetuin-B, and unaffected by zinc. Since the change in elastic modulus is induced by ZP2 cleavage, an automated elastic modulus measurement of oocytes may serve as a novel sensitive, non-destructive, marker-free, and observer-unbiased method for assessing individual oocyte quality.

## Introduction

The number of children conceived by assisted reproductive technique (ART) is continuously rising. From 2008 to 2010, 1 million children were conceived by ART, exceeding 5 million children in the year 2012 and reaching 8 million to date (Ferraretti *et al.* 2012, Dyer *et al.* 2016, Adamsson *et al.* 2019). As the demand for ART grows, the quest to optimize ART intensifies. However, comprehensive and reliable gamete and embryo quality control are still lacking. One important factor of oocyte and embryo quality is the zona pellucida (ZP). The ZP is an extracellular matrix comprising three and four ZP proteins in mice and humans, respectively (Bleil & Wassarman 1980, Bauskin *et al.* 1999, Lefièvre *et al.* 2004). Several studies have shown the importance of a stepwise increase in ZP robustness during fertilization and early embryonic development. This increase in ZP robustness is caused by ovastacin, a metalloproteinase stored in cortical vesicles beneath the oocyte cell membrane (Burkart *et al.* 2012). Pre-ovulation oocytes release small quantities of ovastacin into the perivitelline space to pre-harden the zona pellucida by limited proteolysis of the N-terminal segment of ZP2 called ZP2 cleavage (Ducibella *et al.* 1994, Gahlay *et al.* 2010). Thereby, the ZP gains sufficient robustness to resist mechanical stress during ovulation (Körschgen *et al.* 2017). Nevertheless, the ZP stays permeable for sperm, because the potent ovastacin inhibitor fetuin-B, a liver-derived plasma protein also abundant in follicular fluid, prevents definitive ZP hardening (Dietzel *et al.* 2013). The second step in ZP hardening is induced by sperm entering the oocyte. Sperm entry triggers bulk exocytosis of ovastacin, overwhelming fetuin-B inhibition capacity and thus, triggering definitive ZP hardening (Braden *et al.* 1954, Bleil *et al.* 1981, Burkart *et al.* 2012), which blocks further sperm binding and penetration (Gahlay *et al.* 2010, Avella *et al.* 2014). After fertilization, the ZP protects the embryo on its passage down the oviduct and during preimplantation development. Embryos without intact ZP are readily absorbed by the oviduct epithelium (Modliński 1970, Rankin *et al.* 1999, Rankin & Dean 2000, Floehr *et al.* 2017). In summary, correct ZP processing is crucial for proper fertilization and early embryo development. Accordingly, ZP dysmorphology is associated with diminished pregnancy rate, implantation rate, and live birth rates in animals and humans (Shi *et al.* 2014, Sauerbrun-Cutler *et al.* 2015, Chen *et al.* 2017, Liu *et al.* 2017, Dai *et al.* 2019). For this reason, a quantitative assessment of zona pellucida hardening would help to assess oocyte state, fertility, and thus quality. The aforementioned ZP2 cleavage is associated with increased ZP resistance to heat and proteolysis (Gulyas & Yuan 1985, Lindsay & Hedrick 2004, Dietzel *et al.* 2013, Körschgen *et al.* 2017). However, both measures destroy the oocytes. In human fertility clinics, the oocyte quality is, therefore, judged microscopically by an experienced embryologist. Other methods like time-lapse and polarization microscopy inform about ZP quality and implantation potential, but they fail to provide quantitative or molecular information (Montag *et al.* 2008). Yanez and colleagues showed that the human oocyte developmental potential could be predicted by their mechanical properties (Yanez *et al.* 2016). Mechanical ZP properties vary widely in immature and mature oocytes, after fertilization, during embryo development, and in oocytes of young mice compared to old mice (Murayama *et al.* 2006, Liu *et al.* 2010, Kim & Kim 2013, Andolfi *et al.* 2016). However, a direct link between mechanical hardening and molecular ZP2 cleavage and thus fertility is still lacking.

To address this issue, we studied mechanical properties of MII oocytes with a pre-defined zona status by nanoindentation to correlate the ZP elastic modulus (E-modulus) as a measure of zona hardening with established parameters of proteolytic resistance and ZP2 cleavage. Apart from WT oocytes with normal ZP phenotype, two extreme zona phenotypes were included: fetuin-B deficient (*Fetub^−/−^*) oocytes with premature cleaved ZP2 protein and thus fully hardened ZP (Dietzel *et al.* 2013), and fetuin-B/ovastacin double deficient (*Fetub^−/−^, Astl^−/−^*) oocytes with complete uncleaved ZP2 protein and thus a soft ZP (Floehr *et al.* 2017). The two extreme zona states of *Fetub^−/−^* and *Fetub^−/−^, Astl^−/−^* oocytes thus offer ideal landmarks to quantitatively analyze ZP2 cleavage as a driving force for mechanical ZP hardening.

## Materials and methods

### Ethical approval

All animal experiments were in accordance with the German Animal Welfare law and approved by the State Governments in North-Rhine Westphalia. Maintenance, handling, and treatment of the mice were performed according to the Federation for Laboratory Animal Science Associations (FELASA) recommendations.

### Mouse oocytes and *in vitro* fertilization

C57BL/6 fetuin-B WT (*Fetub^+/+^*, *n* = 25) and fetuin-B deficient (*Fetub^−/−^*, *n* = 14) female mice with identical genetic background were used as well as fetuin-B/ovastacin double deficient (*Fetub^−/−^, Astl^−/−^*, *n* = 54) females (FVB and C57BL/6 mixed genetic background). Oocytes were isolated as described (Dietzel *et al.* 2013). To examine MII oocytes individually, cumulus cells surrounding the oocytes were digested with 0.16 mg/mL hyaluronidase for 3 min (H4272, Sigma). Afterward, oocytes were washed several times in 150 µL EmbryoMax^®^ advanced KSOM embryo medium (MR 101-D, Merck).

In the case of IVF, hyaluronic digestion of cumulus cells was omitted. Sperm of C57BL/6 fetuin-B WT (*Fetub^+/+^*, *n* = 5, 12–17 weeks old) mice was collected as already described in Dietzel *et al.* (2013) and directly mixed with cumulus-oocyte complexes. Twenty-four hours after insemination, the number of two-cell embryos was evaluated and separated for E-modulus measurement and ZP2 analysis. Two-cell embryos for blastocyst development evaluation were kept in KSOM medium until 5 days post-fertilization.

### Forced zona pellucida hardening

Zona pellucida hardening following ovastacin and zinc treatment was assessed using *Fetub^−/−^, Astl^−/−^* oocytes. Oocytes from cumulus-oocyte complexes of two to three females were pooled for each experiment, treated with hyaluronic acid, and transferred to HTF-medium. Recombinant pro-ovastacin was proteolytically activated by human plasmin (HCPM-0140, Haematologic Technologies, Essex Junction, USA) at a molar ratio of 10:1 (Karmilin *et al.* 2019) and added to the oocytes. For inhibition experiments, recombinant fetuin-B proteins were mixed immediately after the addition of activated ovastacin at a molar ratio of 2:1. Oocytes were incubated for 1 h at 37°C and 5% CO_2_. Afterward, oocytes were washed four times in KSOM medium, and E-modulus measurement was performed. To evaluate the effect of zinc on ZP hardening *Fetub^−/−^, Astl^−/−^* oocytes were collected in 300 µL HTF after cumulus digestion and then randomly divided into KSOM medium without or with the addition of 50 and 500 µM ZnSO_4,_ respectively. The oocytes were washed four times in respective medium and then incubated for 1 h at 37°C and 5% CO_2_.

### Elastic modulus measurements

Oocytes or two-cell embryos of different genetic backgrounds (*Fetub^+/+^*; *Fetub^−/−^*; *Fetub^−/−^, Astl^−/−^*) were positioned on 60 µm polyethylene terephthalate meshes (pluriStrainer, pluriSelect, Leipzig, Germany) in KSOM medium for elastic modulus (E-modulus) evaluation. The E-modulus was determined by using a nanoindentation technology (Piuma, Optics11 Life, Amsterdam, Netherlands) with a spherical probe (25 µm tip radius, 0.05 N/m spring constant of cantilever) under microscopic visual control (Dmi1, Leica Microsystems). Oocyte integrity was ensured by keeping them in KSOM medium at 37°C. Integrity criteria were fulfilled when oocytes had a uniform ooplasm without fragment and vacuoles and were surrounded by a uniform zona pellucida structure. Finding surface procedure was followed by indentation-depth controlled measurement consisting of loading at 1 µm/s to 4000 nm indentation depth, holding for 1 s and unloading for 2 s. All oocytes were measured at least three times. Data analysis was done by DataViewer software (Optics11 Life) by fitting the load-indentation curve up to 4000 nm to a Hertz model to calculate the Youngs’s modulus of elasticity (Pa). Data were excluded when indentation started in contact with the oocyte or in case of unstable immobilization showed by dents in the indentation curve indicating an oocyte movement during the measurement. Immobilization was achieved only by friction between oocyte mesh and without any chemical fixation.

### Zona pellucida digestion

Oocytes of *Fetub^+/+^*, *Fetub^−/−^* and *Fetub^−/−^, Astl^−/−^* mice were isolated as previously described in *mouse oocytes and in vitro fertilization*. Denuded oocytes were incubated in 2.0 mg/mL chymotrypsin (#SLBG281V, Sigma) at a constant temperature of 37°C. To determine the time point at which 50% of the oocytes were zona-free (t50), a micrograph was recorded every 30 s and data points were plotted against the time as described (Dietzel *et al.* 2013).

### Zona pellucida protein 2 immunoblot

Oocytes of *Fetub^+/+^*, *Fetub^−/−^* and *Fetub^−/−^, Astl^−/−^* mice were isolated following hormonal treatment or after E-modulus measurements, diluted in SDS-PAGE loading buffer, and 13–32 oocytes were loaded on a 12.5% w/v polyacrylamide gel under reducing conditions. To ensure that a defined number of oocytes/embryos were loaded onto the gel, the oocytes/embryos were counted using a microscope and immediately transferred to loading buffer and boiled. The complete sample was further used for SDS-PAGE. Full-length and cleaved ZP2 were detected with IE-3 MAB as described (Körschgen *et al.* 2017).

### Production of recombinant proteins

Recombinant mouse pro-ovastacin was produced in baculovirus-transduced high five insect cells (Cuppari *et al.* 2019, Karmilin *et al.* 2019). Recombinant mouse (UniProtKB ID: Q9QXC1), chicken (F1NHT5) as well as mouse fetuin-B point mutant (fetuin-B^D156A^), and truncated fetuin-B (fetuin-B^CY1CY2^) were expressed in ExpiCHO-S cells (Thermo Fisher Scientific) according to manufacturer's specifications as described previously (Karmilin *et al.* 2019). Point mutated fetuin-B (fetuin-B^D156A^) was generated as previously described (Cuppari *et al.* 2019). The truncated fetuin-B^CY1CY2^, consisting only out of the first (CY1) and the second cystatin-like domain (CY2), was produced as shown detailed in Supplementary Fig. 1 (see section on supplementary materials given at the end of this article).

## Results

### Nanoindentation measures oocyte mechanical zona pellucida hardening

**Figure 1:**
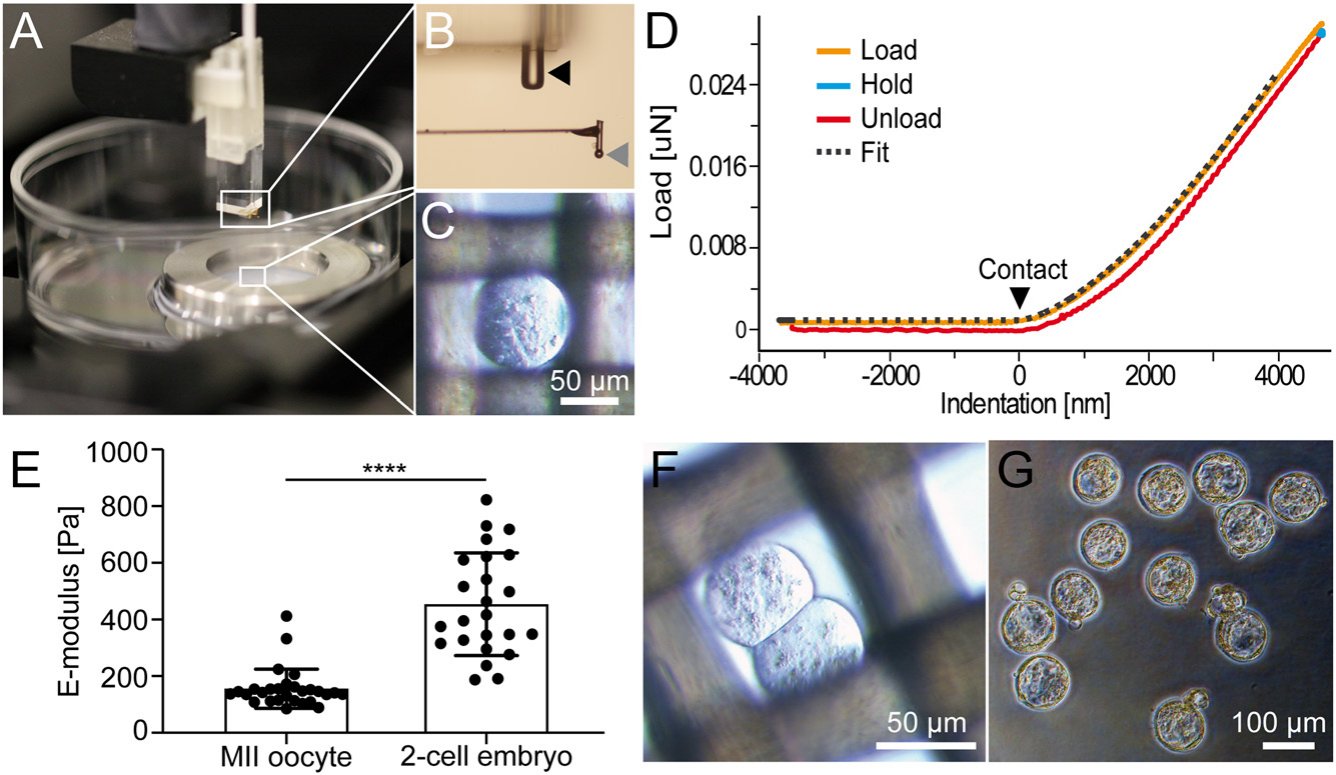
E-modulus measurement of oocytes by nanoindentation. (A) Oocytes were immobilized on 60 µm meshes in a petri dish and kept in medium at constant 37°C. The nanoindenter instrument with a cantilever probe for elastic force measurement was placed above the petri dish. (B) The enlarged image section shows the cantilever with a spherical probe (grey arrow) with the laser shaft (black arrow) of the indentation device placed above. (C) Oocyte integrity was visually evaluated for each oocyte before and during indentation using an inverted microscope. (D) Typical recording of a loading curve divided into load (orange), hold (blue), and unload (red). Fitted curve (grey dashed) was used to calculate the zona pellucida E-modulus by the Hertz model. (E) Determination of the E-modulus of MII oocytes and two-cell embryos of WT mice. Each dot represents the mean of triplicate measurement of an individual oocyte/embryo (F) two-cell embryo on 60 µm mesh and probed by nanoindentation successfully developed into (G) blastocysts, partially hatched, 4 days after indentation. Mann–Whitney test, ****P  < 0.0001. Bars depict the mean ± s.d.

Using a nanoindenter instrument with a lever and a cantilever probe for electronic measurement of mechanical force we measured the E-modulus of WT oocytes within 15 min of harvest. Figure 1A shows the setup with the lever above a petri dish containing the oocytes and a magnification of the cantilever in Fig. 1B. Denuded MII oocytes were immobilized on a mesh (60 µm grid size) without additional chemical fixation and kept under visual integrity control in KSOM medium at 37°C throughout the measurements for up to 60 min (Fig. 1C). Figure 1D shows typical recordings of a load curve from 0 nm deflection to the maximum indentation of 4000 nm into the surface of the ZP. The linear increase of the load curve during the indentation of the ZP confirmed 4000 nm as a depth sufficient for unambiguous E-modulus determination. The recordings were used to calculate the ZP E-modulus based on the Hertz model, which is implemented in the instrument software.

We measured untreated WT mouse oocytes and two-cell embryos, which represent physiological starting- and endpoints of ZP hardening. Figure 1E demonstrates that fertilization of WT oocytes increased the E-modulus from 155 ± 69 Pa to 454 ± 181 Pa (*n* = 28 vs *n* = 24, *P*  < 0.0001) reflecting the physiological ZP hardening after fertilization. Of note, the indentation of 4000 nm into the ZP did not harm the oocytes and did not negatively influence further embryo development. Remarkably, 96% of the two-cell embryos probed by nanoindentation (Fig. 1F, *n* = 23) successfully developed into blastocysts within 5 days post-fertilization (Fig. 1G). In comparison, 66% (*n* = 63) non-indented embryos developed into blastocysts (Supplementary Fig. 2).

### Range of zona pellucida hardening, determined by the extreme ZP states in fetuin-B and ovastacin-deficient mouse models

**Figure 2:**
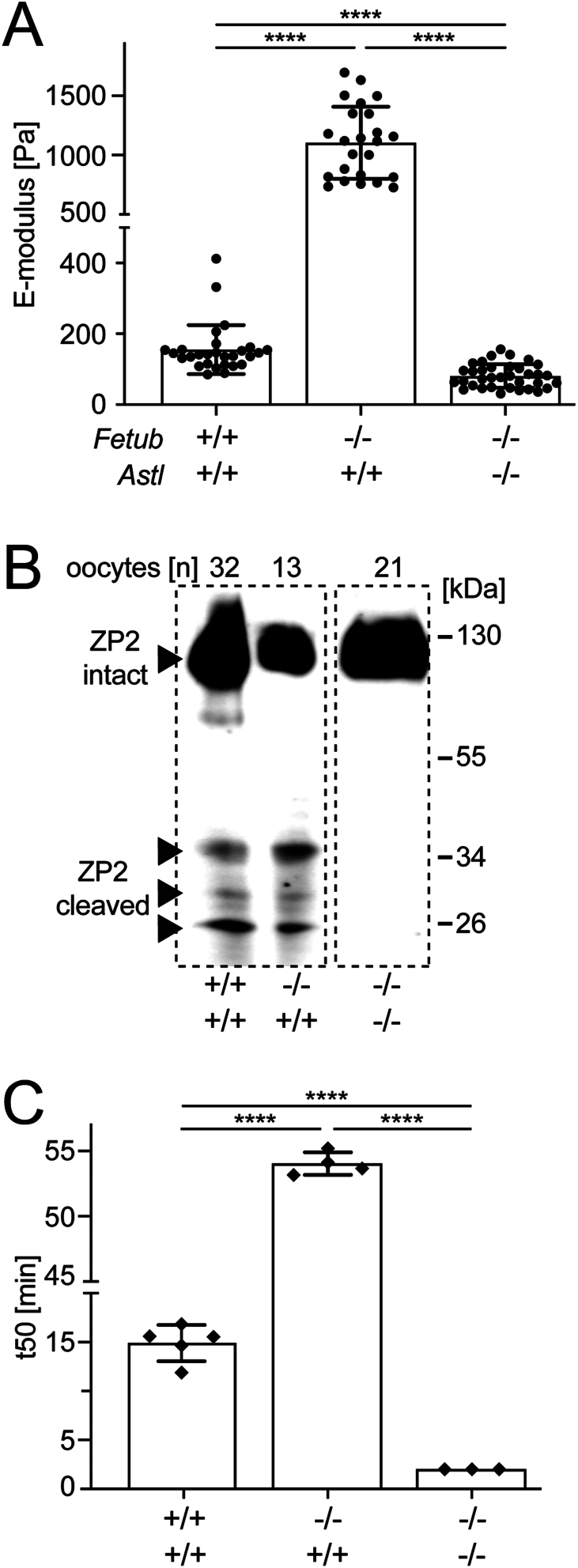
Range of zona pellucida hardening, determined by the extreme states in fetuin-B and ovastacin deficient mouse models with cleaved and uncleaved ZP2, respectively. (A) Determination of the E-modulus of MII oocytes from WT (*Fetub^+/+^, Astl^+/+^*), fetuin-B deficient (*Fetub−/−, Astl^+/+^*) and fetuin-B/ovastacin double deficient (*Fetub−/−, Astl−/−*) mice. Each dot represents the mean of triplicate measurement of one oocyte. (B) Zona pellucida protein 2 (ZP2) immunoblot of nanoindented oocytes from (A). Note that the number of oocytes analyzed ranged from 13 to 32 and, therefore, the relative amount of ZP2 cleavage products is highest in *Fetub−/−, Astl^+/+^* oocytes. (C) ZP digestion times (t_50_) of *Fetub^+/+^, Astl^+/+^; Fetub−/−, Astl^+/+^ and Fetub−/−,* Astl*−/−* oocytes. The t_50_ value equals the time required for 50% of the oocytes to become zona-free following chymotrypsin treatment. Every dot represents one experiment performed with at least ten oocytes. (A) Kruskal–Wallis test, (C) One-way ANOVA, ****P  < 0.0001. Bars depict the mean ± s.d.

Fetuin-B single and fetuin-B/ovastacin double deficient oocytes are representative for infertile and highly fertile oocytes as shown in previous studies with 0% and 95 ± 1% IVF rate, respectively (Dietzel *et al.* 2013, Floehr *et al.* 2017). These two genetic models were used as extreme states of the zona pellucida with cleaved ZP2 and intact ZP2 protein, resulting in a hard and a soft ZP, respectively. Figure 2A shows that the E-modulus of oocytes from these genotypes fully reflects the expected phenotypes. Compared to WT oocytes (155 ± 69 Pa), fetuin-B deficient oocytes (*Fetub^−/−^*, *n* = 24) showed significantly higher E-modulus at 1104 ± 304 Pa (*P*  < 0.0001) reflecting the premature ZP hardening of *Fetub^−/−^* oocytes reported previously (Dietzel *et al.* 2013). These measurements justify the denomination of 'zona pellucida hardening' as they clearly show increased mechanical stiffness of hardened ZP, which was previously merely inferred from surrogate measurements like sensitivity to chymotryptic zona degradation or ZP2 cleavage by immunoblotting. Conversely, fetuin-B/ovastacin double deficient (*Fetub^−/−^, Astl^−/−^*, *n* = 35) oocytes were readily distinguishable from WT by their 'softness' and low E-modulus of 85 ± 33 Pa oocytes (*P*  < 0.0001). The indentation measurements underscored that a soft ZP was associated with the absence of the proteinase ovastacin.

Next, we analyzed if increased E-modulus was associated with increased ZP2 cleavage, the definitive molecular event of ZP hardening. To this end, we analyzed the cleavage status of ZP2 in MII oocytes (*Fetub^+/+^*, *Fetub^−/−^* and *Fetub^−/−^, Astl^−/−^*) by immunoblot. Figure 2B illustrates that ZP2 cleavage was tightly associated with the hardening status of the ZP shown in Fig. 2A. Antibody IE-3 directed against the N-terminal portion of ZP2 detected full-length intact ZP2 at 120 kDa in all oocytes. Oocytes lacking ovastacin (*Fetub^−/−^, Astl^−/−^*) only had 120 kDa full-length ZP2 protein indicating no ZP2 cleavage at all (Fig. 2B). In both *Fetub^−/−^, Astl^+/+^* and *Fetub^+/+^, Astl^+/+^* oocytes, the signal of ZP2 cleavage fragments (at 26 – 34 kDa) were clearly visible. In *Fetub^−/−^, Astl^+/+^* oocytes, the ratio of cleaved ZP2 to the intact form was higher than in *Fetub^+/+^, Astl^+/+^*oocytes. This supports previous publications showing that mature WT (*Fetub^+/+^, Astl^+/+^*) oocytes contain already cleaved ZP2 protein (Dietzel *et al.* 2013, Körschgen *et al.* 2017) and *Fetub^−/−^* oocytes have undergone considerably more ZP2 cleavage leading to premature hardening of the ZP and thus complete infertility in these mice.

Historically, the ZP state was assessed by enzymatic ZP digestion. The time point at which half of a population of oocytes had their ZP completely digested, by for example chymotrypsin, was defined as t_50_, and taken as a measure of ZP hardness. The longest t_50_ time was measured for *Fetub^−/−^* oocytes (54 ± 1 min), while WT (15 ± 2 min) and in particular, *Fetub^−/−^, Astl^−/−^* double deficient oocytes (<2 min) had significantly shorter t_50_ values (Fig. 2C). A comparison of all three methods shows that E-modulus measurements have increased sensitivity compared to ZP2 immunoblotting or ZP digestion analysis. ZP hardening of *Fetub^−/−^* oocytes determined by both ZP2 immunoblot and ZP digestion analysis resulted in a tripling of the reading signal compared to WT oocytes (15 ± 2 vs 54 ± 1 min). However, using the E-modulus a seven-fold increase was observed (155 ± 68 Pa vs 1100 ± 304 Pa).

### Nanoindentation as quantification tool for ZP hardening in individual oocytes

**Figure 3:**
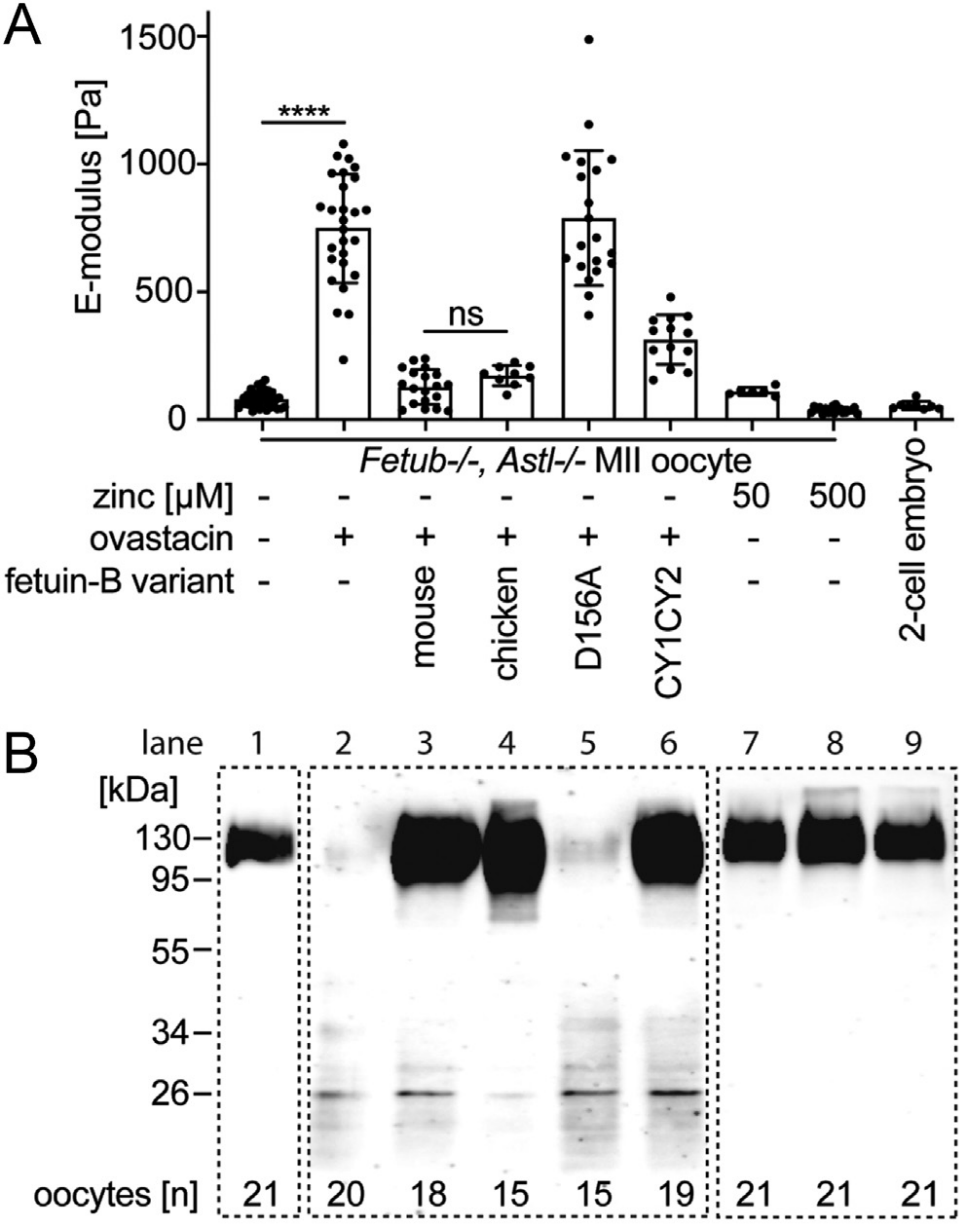
Zona pellucida hardening was induced in *Fetub−/−, Astl−/−* oocytes by exogenous ovastacin and inhibited by mammalian and avian fetuin-B. (A) Determination of the E-modulus of MII oocytes or two-cell embryos from fetuin-B/ovastacin double deficient (*Fetub−/−, Astl−/−*) mice after treatment with recombinant mouse ovastacin and recombinant fetuin-B (mouse, WT mouse fetuin-B; chicken, WT chicken fetuin-B; D156A, mutant mouse fetuin-B^D156A^; CY1CY2, truncated fetuin-B^CY1CY2^) or zinc. Each dot represents the mean of triplicate measurement of one oocyte. Mann–Withney test, ****P  < 0.0001; ns, not significant . Bars depict the mean ± s.d. (B) Zona pellucida protein 2 (ZP2) immunoblot of nanoindented oocytes and embryos from (A).

Ovastacin release from secretory granula of oocytes is required for subsequent ZP2 cleavage and ZP hardening. Accordingly, Fig. 3 shows only soft zona and intact ZP2 in untreated *Fetub^−/−^, Astl^−/−^* oocytes and fertilized two-cell embryos (Fig. 3A and B, lane 1 and 9, respectively). To verify nanoindentation as quantification tool for ZP hardening soft *Fetub^−/−^, Astl^−/−^* oocytes were treated with recombinant mouse ovastacin to induce ZP hardening artificially. Indeed, recombinant ovastacin significantly increased the E-modulus of *Fetub^−/−^, Astl^−/−^* oocytes from 81 ± 33 to 753 ± 215 Pa, *n* = 35 vs *n* = 27, *P*  < 0.0001 (Fig. 3A, lane 1, 2). Moreover, the result indicated that forced hardening by exogenous ovastacin mimicked uninhibited endogenous ovastacin in fetuin-B deficient oocytes (Fig. 2A). Notably, exogenous ovastacin completely abrogated the intact ZP2 signal indicating complete proteolysis (Fig. 3B, lane 2). In comparison in *Fetub^−/−^* oocytes with premature ZP hardening, ZP2 cleavage was incomplete (Fig. 2B) indicating that partial ZP2 cleavage is sufficient for efficient ZP hardening. This hypothesis is supported by the observation that exogenous ovastacin does not increase the E-modulus of premature hardened *Fetub^−/−^* oocytes (Supplementary Fig. 3).

Next, we analyzed if forced ZP hardening triggered by exogenous ovastacin can be inhibited by fetuin-B. Figure 3, lane 3 shows that recombinant mouse fetuin-B completely abrogated ZP hardening and ZP2 cleavage by exogenous ovastacin, underscoring that fetuin-B and ovastacin are a highly conserved proteinase-inhibitor couple. Remarkably, chicken fetuin-B inhibited mouse ovastacin activity comparable to the mouse ortholog (174 ± 40 and 128 ± 69 Pa, *P*  = 0.0775) indicating evolutionary conservation of astacin-like proteinase inhibition by fetuin-B in non-mammalian vertebrates (Fig. 3, lane 4). Accordingly, the ovastacin inhibition motif of fetuin-B, ^154^CPDCP^158^ (nomenclature for mouse fetuin-B (Cuppari *et al.* 2019, Guevara *et al.* 2019)), is highly conserved in chicken fetuin-B (Supplementary Fig. 4). We used nanoindentation to test the importance of this region for the inhibition of ovastacin by adding mutant mouse fetuin-B^D156A^ protein containing a single-point mutation. Notably, mouse fetuin-B^D156A^ protein failed to inhibit ZP hardening and ZP2 fragmentation triggered by exogenous recombinant ovastacin. Instead, the E-modulus was high (794 ± 266 Pa, Fig. 3A, lane 5) and ZP2 cleavage was complete (Fig. 3B, lane 5). The equivalent point mutations in human (D153A) and bovine fetuin-B (D154A) also caused a loss of inhibitory power toward ovastacin, further emphasizing the importance of this motif (Supplementary Fig. 5). Our structure–function study predicted that the C-terminal region of fetuin-B should play a negligible role in direct fetuin-B-ovastacin inhibition but could play an important scaffolding role (Cuppari *et al.* 2019, Guevara *et al.* 2019). We tested this prediction and produced mouse fetuin-B comprising two amino-terminal cystatin-like domains CY1 and CY2, but lacking the C-terminal region CTR (fetuin-B^CY1CY2^, Supplementary Fig. 1). Figure 3A, lane 6 shows that the truncated fetuin-B^CY1CY2^ partially inhibited exogenous ovastacin in that the E-modulus was intermediary (315 ± 98 Pa) between full-length mouse fetuin-B (128 ± 69 Pa) and inactive full-length mouse fetuin-B^D156A^ (794 ± 266 Pa). The lack of the full inhibitory potential demonstrates the crucial scaffolding role of the CTR, as it is not involved in the direct protein–protein interaction. Whether or not the inhibitory potential of fetuin-B^CY1CY2^ is sufficient to maintain the fertility of oocytes requires further studies.

A recent publication stated that endogenous zinc sparks as well as exogenous zinc contribute to ZP hardening (Que *et al.* 2017). We measured E-modulus and ZP2 cleavage after adding 50 and 500 µM zinc to *Fetub^−/−^, Astl^−/−^* oocytes in the absence of the metalloproteinase ovastacin and incubated for 1 h (Fig. 3, lane 7, 8). The addition of zinc neither affected the E-modulus and thus ZP hardening nor did it trigger ZP2 cleavage of ovastacin-free oocytes suggesting that zinc did not induce ZP hardening in our experimental setup. In summary, the results show that nanoindentation of single oocytes is suitable for the quantification of the dynamic process of ZP hardening.

## Discussion

In this study, we showed that single-cell nanoindentation of oocytes allows the quantitative analysis of mechanical ZP hardening. ZP2 cleavage by ovastacin was confirmed as the definitive molecular event triggering mechanical ZP hardening. Consequently, an increase in the E-modulus of MII oocytes is associated with ZP2 cleavage, sperm penetration blockade, and thus reflects oocyte fertility. Therefore, we propose the E-modulus of individual oocytes as a potential novel measure for oocyte quality.

Excessive premature ZP hardening results in fertilization failure (Dietzel *et al.* 2013), and lack of hardening leads to implantation failure (Gahlay *et al.* 2010, Burkart *et al.* 2012, Floehr *et al.* 2017). By using two extreme ZP states of inherently hard *Fetub^−/−^* oocytes and inherently soft *Fetub^−/−^, Astl^−/−^* oocytes, we demonstrated for the first time the strong association of ZP2 cleavage and sensitivity toward chymotryptic digestion, to the non-destructive measurement of the E-modulus.

ZP state is one of many factors influencing oocyte and embryo development. Ideally, different analytical methods should be combined to make informed decisions about oocyte and embryo quality. The established non-invasive human oocyte quality assessment method polarization microscopy proved unsuitable for other species including cattle (Koester *et al.* 2011) and mouse (Supplementary Fig. 6). Alternatively, embryo development to blastocysts was judged by time-lapse imaging, yet with ambiguous results regarding the scoring of embryos and predictive value for further development (Lemmen *et al.* 2008, Hlinka *et al.* 2012, Conaghan *et al.* 2013). Established scoring methods are often descriptive and lack quantitative measures, unlike nanoindentation which is sensitive, marker-free, non-destructive, and free of observer bias. E-modulus measurement by nanoindentation could, therefore, be an adjuvant tool in the future to assess fertility and thus the quality of oocytes. This hypothesis is supported by other studies showing that the mechanical properties of oocytes are readily measurable and closely related to vitality (Palermo *et al.* 1996, Ebner *et al.* 2001, Stracuzzi *et al.* 2021). However, further studies have to be conducted to analyze the E-modulus of oocytes as a prognostic marker for fertilization and pregnancy rate. Our data indicate that nanoindentation of up to 4000 nm did not negatively impact later oocyte and embryo development, yet allowed individual assessment of oocyte quality with a dynamic range and diagnostic precision surpassing simple visual inspection of oocytes. Further reduction of indentation depth and thus mechanical perturbation is possible, but may not be required because the mechanical stimulation did not affect blastocysts development. This point merits further study.

Beside ZP2 cleavage, additional factors may influence ZP hardening. A recent hypothesis suggested that post-fertilization defucosylation of the homodimer ZP1 regulates ZP hardening (Nishimura *et al.* 2019). Several proteases and other factors like p75 were suggested to contribute to ZP hardening, but their role during fertilization is still not fully understood (Schmell & Gulyas 1980, Pierce *et al.* 1990, Zhang *et al.* 1992, Huarte *et al.* 1993). Zinc sparks were proposed to contribute to ZP hardening and prevent sperm binding and penetration (Que *et al.* 2017, Tokuhiro & Dean 2018). In our experimental setup, zinc treatment of ovastacin-deficient oocytes did not cause increased ZP stiffness arguing against a role of 'free' zinc in ZP hardening. This observation supports the conclusion that zinc has no ovastacin-independent effects on ZP hardening. However, it has to be noted that BSA-containing media was used in our setup which could have a chelating effect on zinc, or that the E-modulus measurement is too insensitive to measure zinc-induced changes. This point merits further study.

In summary, this study confirms the reliable, continuous, and sensitive quantification of ZP hardening status of individual oocytes by nanoindentation. The E-modulus as the net outcome of the ZP hardening may, therefore, help to refine assisted reproductive technologies in humans, stock, and laboratory animals alike.

## Supplementary Material

Supplementary Figure 1

Supplementary Figure 2

Supplementary Figure 3

Supplementary Figure 4

Supplementary Figure 5

Supplementary Figure 6

## Declaration of interest

The authors C S, W J-D, and J F are named inventors on a patent application of RWTH Aachen University covering the use of nanoindentation technology for oocyte quality control. S Z S, H K, M K, J S, and W S declare no conflict of interest.

## Funding

The work was supported by a grant from Deutsche Forschungsgemeinschaft http://dx.doi.org/10.13039/501100001659 FL1033/1-1.

## Authors contribution statement

C S, W J D, and J F made substantial contributions to conception of the study. C S, S Z S, H K, M K, W J D, J F acquired data and interpretated data. C S, H K, M K, J S, W S, W J D, J F drafted the article or revised it critically for important intellectual content. The final approval of the version to be published was done by all authors.

## References

[bib1] Adamsson G de Mouzon J Chambers G Zegers-Hochschild F Mansour R Ishihara O Banker M Dyer S Kupka M 2019 International Comittee for Monitoring Assisted Reproductive Technologies (ICMART): World Report on assisted reproductive technology 2015. Vienna, Austria: ESHRE.

[bib2] Andolfi L Masiero E Giolo E Martinelli M Luppi S dal Zilio S Delfino I Bortul R Zweyer M Ricci G ***et al***. 2016 Investigating the mechanical properties of zona pellucida of whole human oocytes by atomic force spectroscopy. Integrative Biology: Quantitative Biosciences from Nano to Macro 8 886–893. (10.1039/c6ib00044d) 27476747

[bib3] Avella MA Baibakov B Dean J 2014 A single domain of the ZP2 zona pellucida protein mediates gamete recognition in mice and humans. Journal of Cell Biology 205 801–809. (10.1083/jcb.201404025) 24934154 PMC4068139

[bib4] Bauskin AR Franken DR Eberspaecher U Donner P 1999 Characterization of human zona pellucida glycoproteins. Molecular Human Reproduction 5 534–540. (10.1093/molehr/5.6.534) 10341000

[bib5] Bleil JD Wassarman PM 1980 Structure and function of the zona pellucida: identification and characterization of the proteins of the mouse oocyte’s zona pellucida. Developmental Biology 76 185–202. (10.1016/0012-1606(8090371-1) 7380091

[bib6] Bleil JD Beall CF Wassarman PM 1981 Mammalian sperm-egg interaction: fertilization of mouse eggs triggers modification of the major zona pellucida glycoprotein, ZP2. Developmental Biology 86 189–197. (10.1016/0012-1606(8190329-8) 6793422

[bib7] Braden AWH Austin CR David HA 1954 The reaction of the zona pellucida to sperm penetration. Australian Journal of Biological Sciences 7 391–409. (10.1071/bi9540391) 13219045

[bib8] Burkart AD Xiong B Baibakov B Jiménez-Movilla M Dean J 2012 Ovastacin, a cortical granule protease, cleaves ZP2 in the zona pellucida to prevent polyspermy. Journal of Cell Biology 197 37–44. (10.1083/jcb.201112094) 22472438 PMC3317803

[bib9] Chen T Bian Y Liu X Zhao S Wu K Yan L Li M Yang Z Liu H Zhao H 2017 A recurrent missense mutation in ZP3 causes empty follicle syndrome and female infertility. American Journal of Human Genetics 101 459–465. (10.1016/j.ajhg.2017.08.001) 28886344 PMC5590947

[bib10] Conaghan J Chen AA Willman SP Ivani K Chenette PE Boostanfar R Baker VL Adamson GD Abusief ME Gvakharia M ***et al***. 2013 Improving embryo selection using a computer-automated time-lapse image analysis test plus day 3 morphology: results from a prospective multicenter trial. Fertility and Sterility 100 412.e5–419.e5. (10.1016/j.fertnstert.2013.04.021) 23721712

[bib11] Cuppari A Körschgen H Fahrenkamp D Schmitz C Guevara T Karmilin K Kuske M Olf M Dietzel E Yiallouros I ***et al***. 2019 Structure of mammalian plasma fetuin-B and its mechanism of selective metallopeptidase inhibition. IUCrJ 6 317–330. (10.1107/S2052252519001568) PMC6400186 30867929

[bib12] Dai C Hu L Gong F Tan Y Cai S Zhang S Dai J Lu C Chen J Chen Y ***et al***. 2019 ZP2 pathogenic variants cause in vitro fertilization failure and female infertility. Genetics in Medicine 21 431–440. (10.1038/s41436-018-0064-y) 29895852

[bib13] Dietzel E Wessling J Floehr J Schäfer C Ensslen S Denecke B Rösing B Neulen J Veitinger T Spehr M ***et al***. 2013 Fetuin-B, a liver-derived plasma protein is essential for fertilization. Developmental Cell 25 106–112. (10.1016/j.devcel.2013.03.001) 23562279

[bib14] Ducibella T Duffy P Buetow J 1994 Quantification and localization of cortical granules during oogenesis in the mouse. Biology of Reproduction 50 467–473. (10.1095/biolreprod50.3.467) 8167217

[bib15] Dyer S Chambers GM de Mouzon J Nygren KG Zegers-Hochschild F Mansour R Ishihara O Banker M Adamson GD 2016 International Committee for Monitoring Assisted Reproductive Technologies world report: Assisted Reproductive Technology 2008, 2009 and 2010. Human Reproduction 31 1588–1609. 27207175 10.1093/humrep/dew082

[bib16] Ebner T Yaman C Moser M Sommergruber M Jesacher K Tews G 2001 A prospective study on oocyte survival rate after ICSI: influence of injection technique and morphological features. Journal of Assisted Reproduction and Genetics 18 623–628. (10.1023/a:1013171505702) 11808841 PMC3455248

[bib17] Ferraretti AP Goossens V DeMouzon J Bhattacharya S Castilla JA Korsak V Kupka M Nygren KG Nyboe Andersen AEuropean IVF-Monitoring (EIM) 2012 Assisted Reproductive Technology in Europe, 2008: results generated from European Registers by ESHRE. Human Reproduction 27 2571–2584. (10.1093/humrep/des255) 22786779

[bib18] Floehr J Dietzel E Schmitz C Chappell A Jahnen-Dechent W 2017 Down-regulation of the liver-derived plasma protein fetuin-B mediates reversible female infertility. Molecular Human Reproduction 23 34–44. (10.1093/molehr/gaw068) 27733488

[bib19] Gahlay G Gauthier L Baibakov B Epifano O Dean J 2010 Gamete recognition in mice depends on the cleavage status of an egg’s zona pellucida protein. Science 329 216–219. (10.1126/science.1188178) 20616279 PMC3272265

[bib20] Guevara T Körschgen H Cuppari A Schmitz C Kuske M Yiallouros I Floehr J Jahnen-Dechent W Stöcker W Gomis-Rüth FX 2019 The C-terminal region of human plasma fetuin-B is dispensable for the raised-elephant-trunk mechanism of inhibition of astacin metallopeptidases. Scientific Reports 9 14683. (10.1038/s41598-019-51095-y) 31604990 PMC6789097

[bib21] Gulyas BJ Yuan LC 1985 Cortical reaction and zona hardening in mouse oocytes following exposure to ethanol. Journal of Experimental Zoology 233 269–276. (10.1002/jez.1402330215) 4038732

[bib22] Hlinka D Kaľatová B Uhrinová I Dolinská S Rutarová J Řezáčová J Lazarovská S Dudáš M 2012 Time-lapse cleavage rating predicts human embryo viability. Physiological Research 61 513–525. (10.33549/physiolres.932287) 22881225

[bib23] Huarte J Vassalli JD Belin D Sakkas D 1993 Involvement of the plasminogen activator/plasmin proteolytic cascade in fertilization. Developmental Biology 157 539–546. (10.1006/dbio.1993.1156) 8388818

[bib24] Karmilin K Schmitz C Kuske M Körschgen H Olf M Meyer K Hildebrand A Felten M Fridrich S Yiallouros I 2019 Mammalian plasma fetuin-B is a selective inhibitor of ovastacin and meprin metalloproteinases. Scientific Report 9 1–12. 10.1038/s41598-018-37024-5 PMC6346019 30679641

[bib25] Kim J Kim J 2013 Viscoelastic characterization of mouse zona pellucida. IEEE Transactions on Bio-Medical Engineering 60 569–575. (10.1109/TBME.2012.2230444) 23212311

[bib26] Koester M Mohammadi-Sangcheshmeh A Montag M Rings F Schimming T Tesfaye D Schellander K Hoelker M 2011 Evaluation of bovine zona pellucida characteristics in polarized light as a prognostic marker for embryonic developmental potential. Reproduction 141 779–787. (10.1530/REP-10-0471) 21415090

[bib27] Körschgen H Kuske M Karmilin K Yiallouros I Balbach M Floehr J Wachten D Jahnen-dechent W Stöcker W 2017 Intracellular activation of ovastacin mediates pre-fertilization hardening of the zona pellucida. Molecular Human Reproduction 23 607–616. (10.1093/molehr/gax040) 28911209 PMC5909858

[bib28] Lefièvre L Conner SJ Salpekar A Olufowobi O Ashton P Pavlovic B Lenton W Afnan M Brewis IA Monk M ***et al***. 2004 Four zona pellucida glycoproteins are expressed in the human. Human Reproduction 19 1580–1586. (10.1093/humrep/deh301) 15142998

[bib29] Lemmen JG Agerholm I Ziebe S 2008 Kinetic markers of human embryo quality using time-lapse recordings of IVF/ICSI-fertilized oocytes. Reproductive Biomedicine Online 17 385–391. (10.1016/s1472-6483(1060222-2) 18765009

[bib30] Lindsay LL Hedrick JL 2004 Proteolysis of Xenopus laevis egg envelope ZPA triggers envelope hardening. Biochemical and Biophysical Research Communications 324 648–654. (10.1016/j.bbrc.2004.09.099) 15474476

[bib31] Liu X Fernandes R Jurisicova A Casper RF Sun Y 2010 In situ mechanical characterization of mouse oocytes using a cell holding device. Lab on a Chip 10 2154–2161. (10.1039/c004706f) 20544113

[bib32] Liu W Li K Bai D Yin J Tang Y Chi F Zhang L Wang Y Pan J Liang S 2017 Dosage effects of ZP2 and ZP3 heterozygous mutations cause human infertility. Human Genetics 136 975–985. (10.1007/s00439-017-1822-7) 28646452

[bib33] Modliński JA 1970 The role of the zona pellucida in the development of mouse eggs in vivo. Journal of Embryology and Experimental Morphology 23 539–547. 5473304

[bib34] Montag M Schimming T Köster M Zhou C Dorn C Rösing B van der Ven H van der Ven K 2008 Oocyte zona birefringence intensity is associated with embryonic implantation potential in ICSI cycles. Reproductive Biomedicine Online 16 239–244. (10.1016/s1472-6483(1060580-9) 18284880

[bib35] Murayama Y Mizuno J Kamakura H Fueta Y Nakamura H Akaishi K Anzai K Watanabe A Inui H Omata S 2006 Mouse zona pellucida dynamically changes its elasticity during oocyte maturation, fertilization and early embryo development. Human Cell 19 119–125. (10.1111/j.1749-0774.2006.00019.x) 17257374

[bib36] Nishimura K Dioguardi E Nishio S Villa A Han L Matsuda T Jovine L 2019 Molecular basis of egg coat cross-linking sheds light on ZP1-associated female infertility. Nature Communications 10 3086. (10.1038/s41467-019-10931-5) PMC6626044 31300655

[bib37] Palermo GD Alikani M Bertoli M Colombero LT Moy F Cohen J Rosenwaks Z 1996 Oolemma characteristics in relation to survival and fertilization patterns of oocytes treated by intracytoplasmic sperm injection. Human Reproduction 11 172–176. (10.1093/oxfordjournals.humrep.a019012) 8671181

[bib38] Pierce KE Siebert MC Kopf GS Schultz RM Calarco PG 1990 Characterization and localization of a mouse egg cortical granule antigen prior to and following fertilization or egg activation. Developmental Biology 141 381–392. (10.1016/0012-1606(9090392-v) 1698670

[bib39] Que EL Duncan FE Bayer AR Philips SJ Roth EW Bleher R Gleber SC Vogt S Woodruff TK O’Halloran TV 2017 Zinc sparks induce physiochemical changes in the egg zona pellucida that prevent polyspermy. Integrative Biology: Quantitative Biosciences from Nano to Macro 9 135–144. (10.1039/c6ib00212a) 28102396 PMC5439353

[bib40] Rankin T Dean J 2000 The zona pellucida: using molecular genetics to study the mammalian egg coat. Reviews of Reproduction 5 114–121. (10.1530/ror.0.0050114) 10864856

[bib41] Rankin TL Talbot P Lee E Dean J 1999 Abnormal zonae Pellucidae in mice lacking ZP1 result in early embryonic loss. Development 126 3847–3855. (10.1242/dev.126.17.3847) 10433913

[bib42] Sauerbrun-Cutler MT Vega M Breborowicz A Gonzales E Stein D Lederman M Keltz M 2015 Oocyte zona pellucida dysmorphology is associated with diminished in-vitro fertilization success. Journal of Ovarian Research 8 5. (10.1186/s13048-014-0111-5) 25823613 PMC4355133

[bib43] Schmell ED Gulyas BJ 1980 Mammalian sperme-egg recognition and binding in vitro. specificity of sperm interactions with live and fixed eggs in homologous and heterologous inseminations of hamster, mouse, and guinea pig oocytes. Biology of Reproduction 23 1075–1085. (10.1095/biolreprod23.5.1075) 6781543

[bib44] Shi W Xu B Wu LM Jin RT Luan HB Luo LH Zhu Q Johansson L Liu YS Tong XH 2014 Oocytes with a dark zona pellucida demonstrate lower fertilization, implantation and clinical pregnancy rates in IVF/ICSI cycles. PLoS ONE 9 e89409. (10.1371/journal.pone.0089409) PMC3933533 24586757

[bib45] Stracuzzi A Dittmann J Böl M Ehret AE 2021 Visco- and poroelastic contributions of the zona pellucida to the mechanical response of oocytes. Biomechanics and Modeling in Mechanobiology 20 751–765. (10.1007/s10237-020-01414-4) 33533999 PMC7979617

[bib46] Tokuhiro K Dean J 2018 Glycan-independent gamete recognition triggers egg zinc sparks and ZP2 cleavage to prevent polyspermy. Developmental Cell 46 627.e5–640.e5. (10.1016/j.devcel.2018.07.020) 30122633 PMC6549238

[bib47] Yanez LZ Han J Behr BB Pera RAR Camarillo DB 2016 Human oocyte developmental potential is predicted by mechanical properties within hours after fertilization. Nature Communications 7 10809. (10.1038/ncomms10809) PMC4770082 26904963

[bib48] Zhang X Rutledge J Khamsi F Armstrong DT 1992 Release of tissue-type plasminogen activator by activated rat eggs and its possible role in the zona reaction. Molecular Reproduction and Development 32 28–32. (10.1002/mrd.1080320106) 1515147

