# Supplementary figures and images for: The E-modulus of the oocyte is a non-destructive measure of zona pellucida hardening
Source: Reproduction. 2021 Jul 28;162(4):259–66. doi: 10.1530/REP-21-0122 (PMC8494380; doi:10.1530/REP-21-0122)

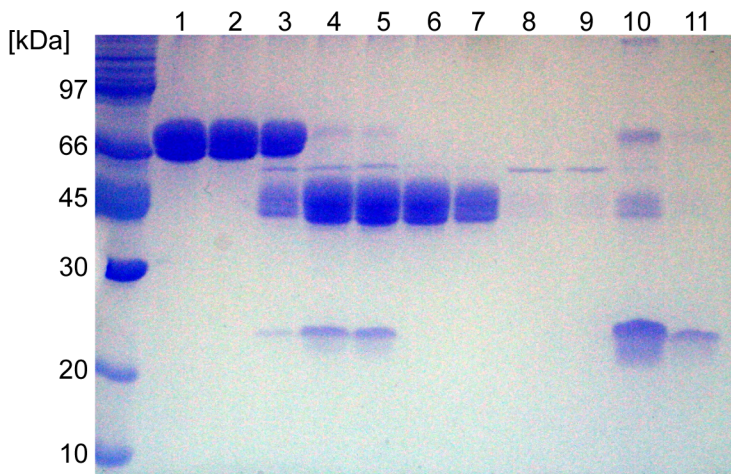

Supplement: Supplementary Figure 1 [file supplementary_figure_1.pdf]

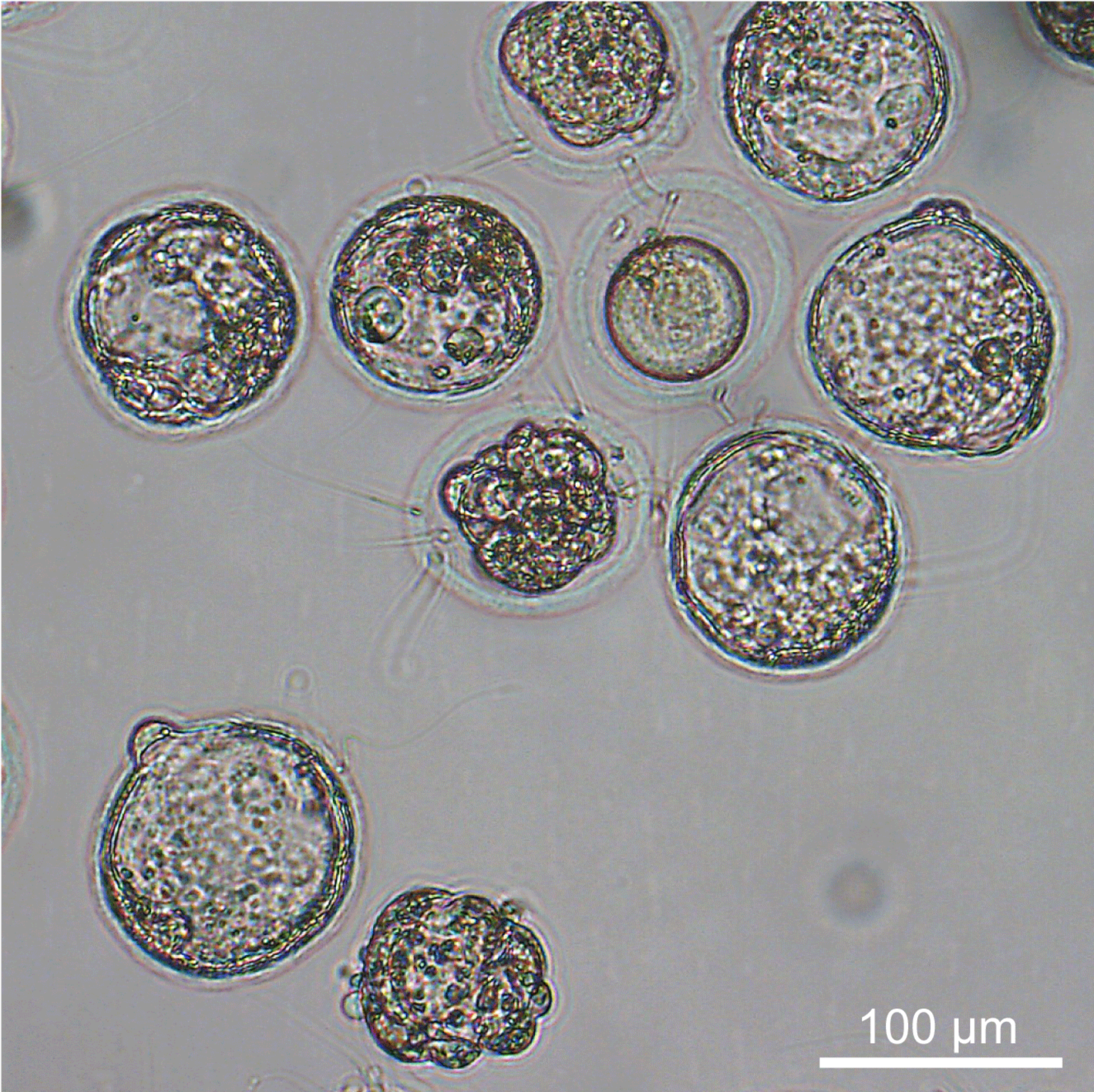

100  $\mu\text{m}$

Supplement: Supplementary Figure 2 [file supplementary_figure_2.pdf]

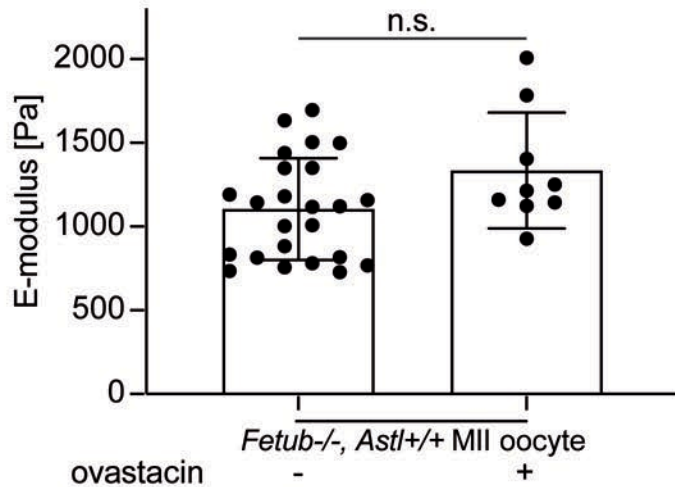

Supplement: Supplementary Figure 3 [file supplementary_figure_3.pdf]

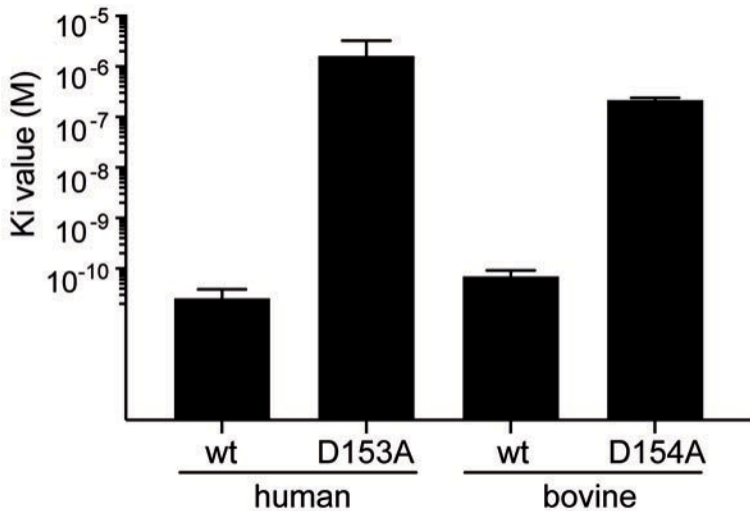

Supplement: Supplementary Figure 5 [file supplementary_figure_5.pdf]

Brightfield

Polarization filter

**A**

*Fetub*<sup>-/-</sup>

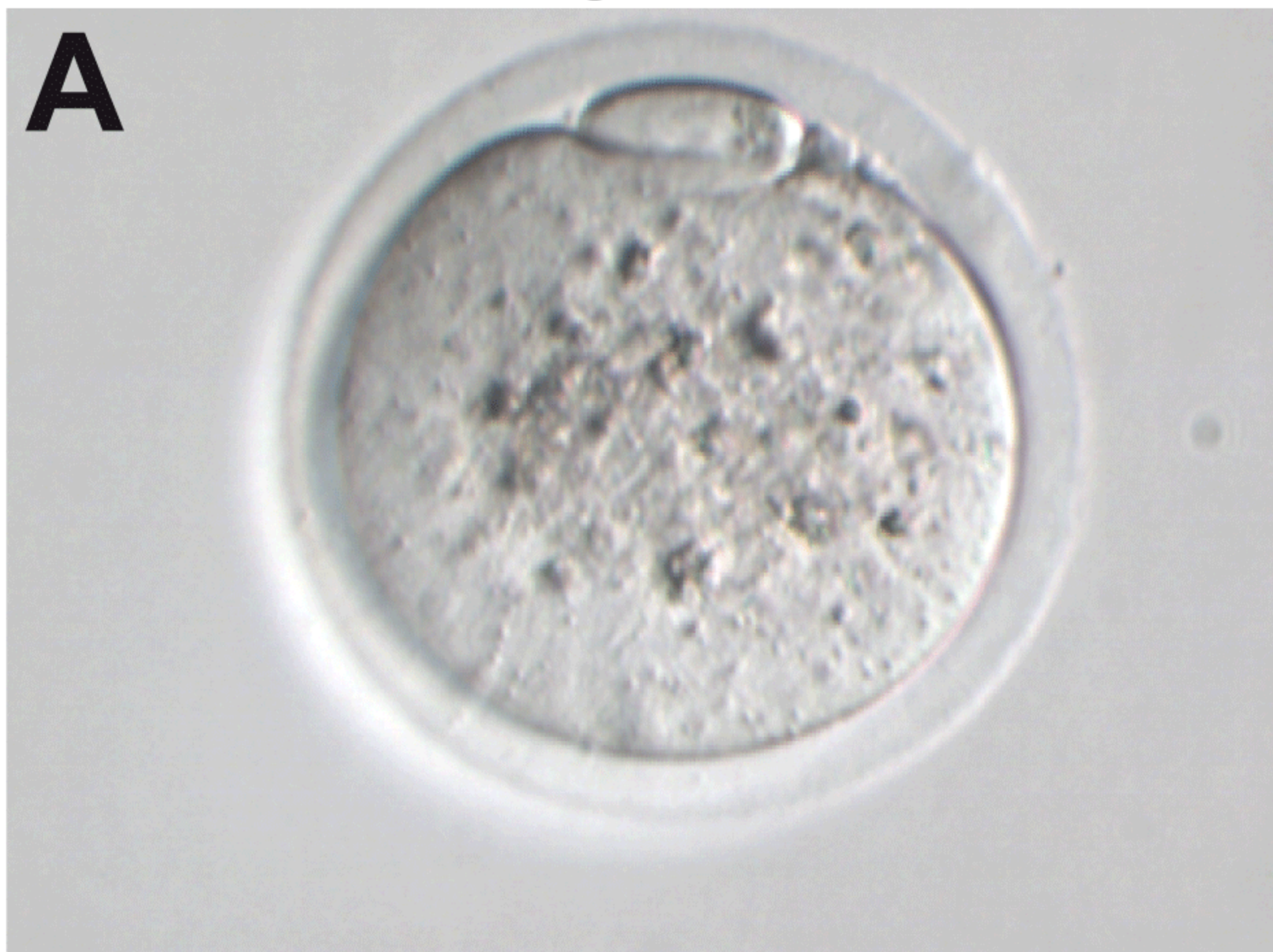

**B**

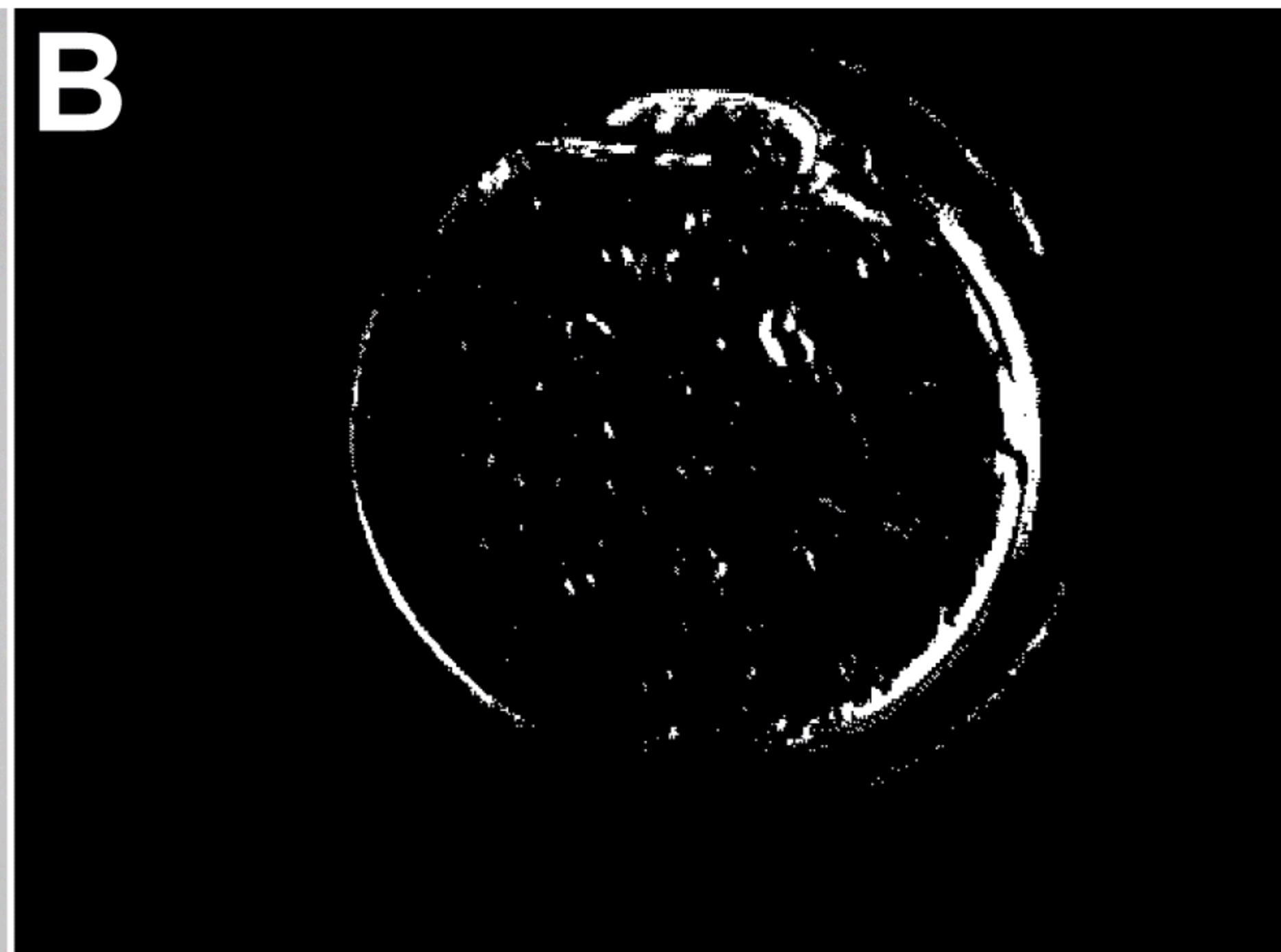

**C**

*Fetub*<sup>-/-</sup>, *Astl*<sup>-/-</sup>

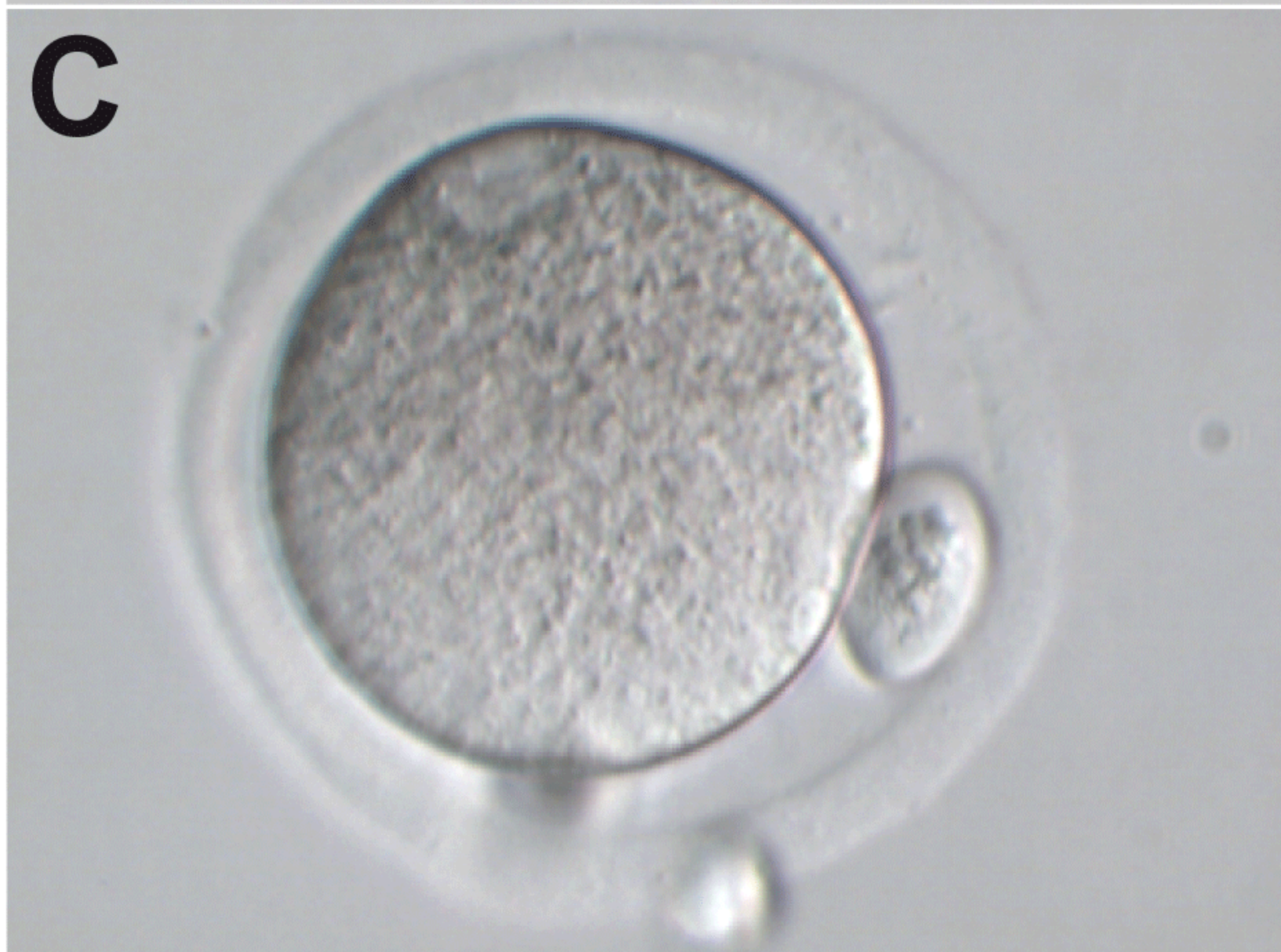

**D**

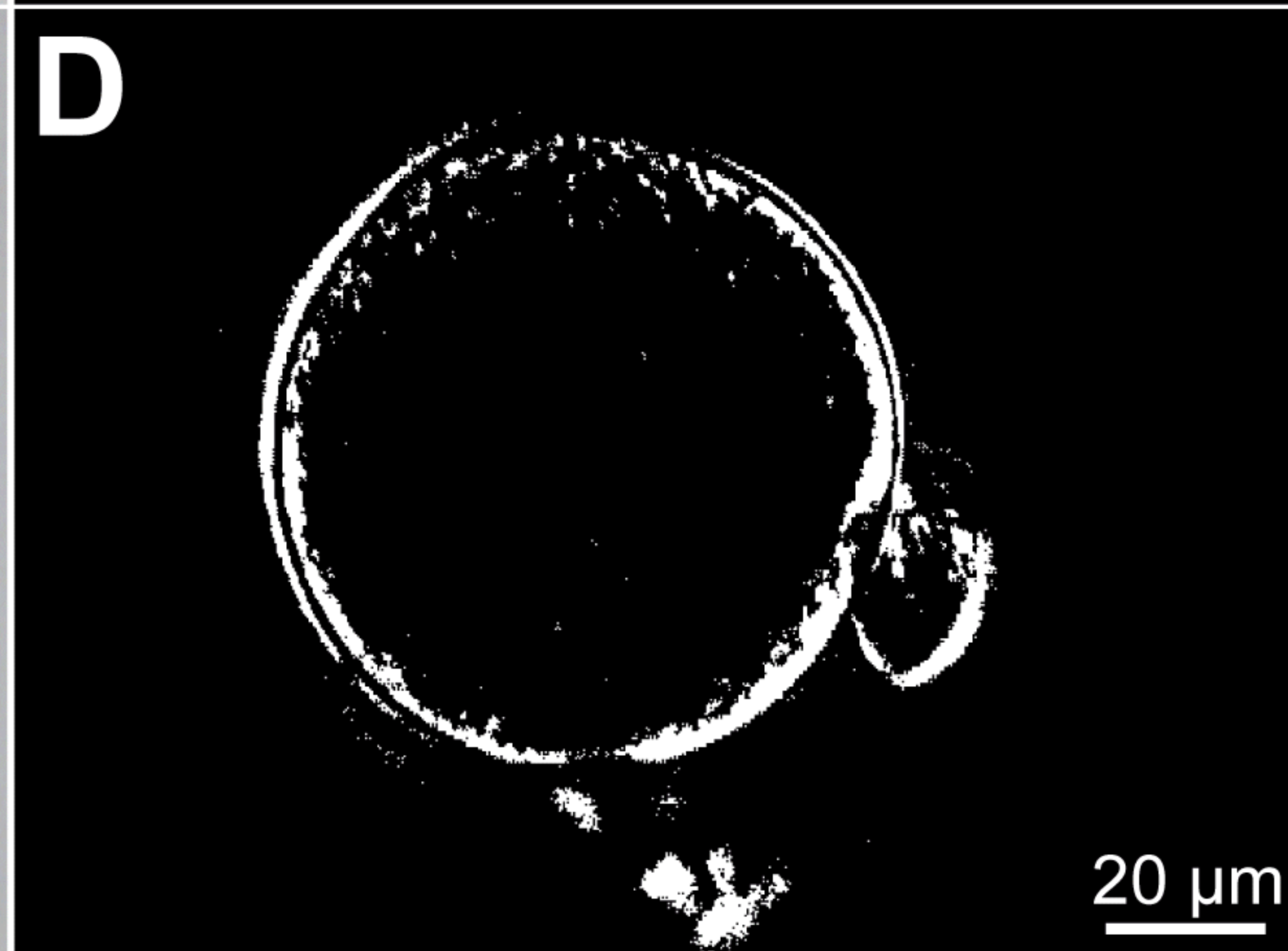

20 μm

Supplement: Supplementary Figure 6 [file supplementary_figure_6.pdf]
